# Views of advance care planning in older hospitalized patients following an emergency admission: A qualitative study

**DOI:** 10.1371/journal.pone.0273894

**Published:** 2022-09-01

**Authors:** Anna-Maria Bielinska, Gehan Soosaipillai, Julia Riley, Ara Darzi, Catherine Urch, Stephanie Archer

**Affiliations:** 1 Department of Surgery and Cancer, Imperial College London, London, United Kingdom; 2 Imperial College Healthcare NHS Trust, London, United Kingdom; 3 The Royal Marsden NHS Foundation Trust, London, United Kingdom; 4 Department of Public Health and Primary Care, University of Cambridge, Cambridge, United Kingdom; 5 Department of Psychology, University of Cambridge, Cambridge, United Kingdom; University of Bristol, UNITED KINGDOM

## Abstract

**Background:**

There is increasing evidence of the need to consider advance care planning (ACP) for older adults who have been recently admitted to hospital as an emergency. However, there is a gap in knowledge regarding how to facilitate ACP following acute illness in later life.

**Aim/Objectives:**

To explore the perceived impact of ACP on the lives of older persons aged 70+ who have been acutely admitted to hospital.

**Method:**

Semi-structured qualitative interviews were conducted with older adults aged 70+ who were admitted to hospital as an emergency. Thematic analysis was enhanced by dual coding and exploration of divergent views within an interdisciplinary team.

**Results:**

Twenty participants were interviewed. Thematic analysis generated the following themes: (1) Bespoke planning to holistically support a sense of self, (2) ACP as a socio-cultural phenomenon advocating for older persons rights, (3) The role of personal relationships, (4) Navigating unfamiliar territory and (5) Harnessing resources.

**Conclusion:**

These findings indicate that maintaining a sense of personal identity and protecting individuals’ wishes and rights during ACP is important to older adults who have been acutely unwell. Following emergency hospitalization, older persons believe that ACP must be supported by a network of relationships and resources, improving the likelihood of adequate preparation to navigate the uncertainties of future care in later life. Therefore, emergency hospitalization in later life, and the uncertainty that may follow, may provide a catalyst for patients, carers and healthcare professionals to leverage existing or create new relationships and target resources to enable ACP, in order to uphold older persons’ identity, rights and wishes following acute illness.

## Introduction

As approximately one in five hospitalized adults aged 70 and over are in the last year of life [1], an emergency hospitalization for an older adult may provide an opportunity to discuss future care preferences [1–3]. Advance care planning (ACP) has been described as an opportunity for individuals to discuss future care plans with their healthcare provider [4]. ACP is a spectrum of possible discussions, including future care planning, urgent care planning and end-of-life care planning [5]. Future care planning considers person-centred care for those with several years prognosis, including health, well-being, and disease management [3, 6]. Urgent care planning is mostly targeted to people with a 1–2-year life expectancy, considering medical care, prognosis, treatment limitations, and may include cardio-pulmonary resuscitation decisions [7]. End-of-life care plans focus on providing patient-centred care for people with a life expectancy a year or less, and may discuss place of care, preferred place of death and terminal wishes [8].

There is increasing awareness of the need for ACP in hospital care, particularly following an acute admission [1–3, 9]. ACP has been shown to improve care, particularly when providing end-of-life care that is concordant with wishes by facilitating decision making [7, 10, 11]. Healthcare professionals are encouraged to engage with ACP as part of their practice [4, 5, 12]. Despite this, evidence suggests that the prevalence of ACP in the acute hospital environment is low, with limited uptake of ACP in older and seriously ill inpatients [13, 14]. Furthermore, there is a knowledge gap in how to incorporate ACP in the acute, time-pressured hospital environment [15].

To better support older adults who have been acutely hospitalized, there is a need to understand the barriers and facilitators to the uptake and completion of ACP. Qualitative studies are fundamental in understanding topics such as these [15–17], but there is a lack qualitative research exploring views of ACP within this cohort, including the perceived impact on older persons and how ACP can be enacted for older persons in this context. This study investigates the views of ACP in older patients aged 70 and over who have been admitted to hospital following an emergency. The study specifically addresses how ACP can impact on the individual lives of older persons, including how ACP can be enacted for older individuals from societal and health systems perspectives. Therefore, the aim of this study is to explore the perceived impact of ACP on the lives of older persons aged 70 and over who have been admitted to hospital as an emergency.

## Methods

### Design

Qualitative interview study with patients aged 70 and over who had been admitted to hospital via an emergency admission.

### Participants and recruitment

The study was carried out at a large urban teaching hospital with an emergency department. Potential participants were identified from a random computer-generated list of patients aged 70 and over who had been admitted overnight as an emergency to the hospital. This sampling method was used to recruit a broad range of patients quickly in a systematic fashion. This approach helped to invite patients with a range of reasons for admission and with a range of demographic characteristics, rather than relying on selecting potential participants from a convenience sample of those who had not yet been moved to different parts of the hospital from the emergency department (i.e. to short and long-stay wards in various medical and surgical specialties).

The age criterion of 70+ years has been used for this study sample. Although there is no universally accepted chronological cut-off for later life, studies have used 70+ as a cut-off in cancer [18] and non-cancer care research in later life [19]. The UK Office for National Statistics considers 70+ as an age cut-off for later life -“age 70 the new age 65” [20]. Furthermore, there are self-care and healthy ageing strategies targeted towards the over 70s in England [21].

Inclusion and exclusion criteria were pragmatically chosen to reflect broad population of older adults who had been admitted acutely to hospital. Patients were included if they were aged 70 years and had been admitted for at least one overnight stay following an emergency admission. Patients of any diagnosis were eligible, provided they were sufficiently clinically stable to give an in-depth interview in English and give informed consent. Patients were excluded if they lacked capacity (defined by the Mental Capacity Act, 2005 [22]) were subsequently admitted to a high dependency or intensive care unit or were believed to have a terminal prognosis (diagnosed as actively dying by the inpatient care team, including persons who had an end-of-life care plan in place which was being actively followed, and those believed to have a prognosis of days to weeks).

The inpatient clinical team (e.g. nurse or doctor) assessed whether patients were stable for interview, initially asking potential participants whether they agreed to be approached by a researcher to be invited to the study. Patients were offered a participant information sheet to read; if willing to participate they gave written informed consent obtained by the first author (A-MB). Participants were encouraged to reflect on ACP generally and were not required to discuss their individual situation unless they volunteered to do so. Participants were interviewed on the hospital ward either by the hospital bed space or in a separate quiet room if preferred. The interviews were conducted at quieter times of day to avoid inpatient ward rounds or medication administration rounds. Participants had the option to discontinue the interview at any stage if they wished.

### Materials and procedure

Interviews were conducted in person within the hospital inpatient setting by A-MB, a female doctor outside the patient’s clinical care team with a specialist interest in ACP. Participants were aware of the interviewer’s academic interest in ACP. Interviews were based on a semi-structured interview schedule developed with a group of patients and carers interested in ACP [23] and informed by published literature [6, 11, 15, 17, 24, 25].

The semi-structured interview explored older adults’ views of ACP. The topic guide explored the: preferred language of ACP, the content of ACP (including a list of possible topics modified from Boyd et al. [6]), the advantages, disadvantages and attitudes to sharing advance care plans. To promote discussion, participants were also shown an information leaflet from Coordinate My Care, an Electronic Palliative Care Coordination System (EPaCCS), of an older patient with advanced symptomatic cancer with a CMC plan who had attended hospital as an emergency.

Interviews were digitally audio-recorded (average length of 34 minutes) and transcribed verbatim. Field notes were also taken by the researcher (A-MB). Data collection continued until thematic saturation was achieved.

### Ethical approval

Research ethics approval was granted by the Hampstead Research Ethics Committee (Ref: 16/LO/1798).

### Data analysis

Interview transcripts were analysed via the six-phase process of thematic analysis [26]. Thematic analysis has been used elsewhere in qualitative research focusing on ACP in a variety of settings [2, 3, 27–29]. Transcripts were read repeatedly, with notes created regarding initial possible codes and thereafter systematic line-by-line manual coding and searching for sub-themes and themes, with further notes on data patterns (by A-MB). Candidate subthemes and themes were reviewed at the level of coded data extracts (by AM-B). The sub-themes and themes generated were further reviewed and refined at the level of a thematic map and named by A-MB and discussed with SA (a Health Psychologist with expertise in qualitative methodology) and CEU (a Professor and Consultant in Palliative Medicine), as part of a multi-disciplinary team. Two interviews were second-coded by another researcher (GS), with findings discussed in a face-to-face meeting with A-MB, revealing high convergence. A-MB produced the report using examples from the dataset to demonstrate themes and understanding, together with discussion with SA and CEU. Throughout the analysis, authors discussed findings to resolve discrepancies.

## Results

In total, 20 patients were recruited into the study; 13 were male and 7 were female. There were no dropouts following consent. Participants were aged between 70 and 93 years. The majority lived alone at home (12/20). The participants’ characteristics are summarised in Table 1.

**Table 1:** Participant characteristics.

Participant number	Age	Gender	Social background	Known current cancer diagnosis	Reason for emergency admission
1	89	Female	Lives alone	No	Asthma exacerbation
2	79	Female	Lives with husband who is main carer	No	Side-effects of analgesia for severe joint pain
3	91	Female	Lives alone with visits from carers	No	Fall
4	74	Male	Lives with wife	Yes -bladder cancer	Complications relating to cancer
5	70	Female	Lives alone	No	Fall
6	83	Female	Lives alone	No	Heart failure
7	85	Male	Lives with wife	No	Nosebleed
8	77	Female	Lives alone	Yes -metastatic sarcoma	Generalised deterioration
9	77	Female	Lives with son	No	Chest infection
10	84	Male	Lives alone, with visits from carers	No	Complications related to rheumatoid arthritis
11	78	Male	Lives in care home	No	Complications related to Parkinson’s disease
12	76	Female	Lives alone	Yes -ovarian cancer	Renal complications
13	76	Female	Lives with son	No	Chest infection on background of COPD
14	75	Female	Lives alone	No	Severe leg oedema
15	77	Male	Lives with wife	No	Urinary tract infection and internal bleeding
16	81	Male	Lives alone	No	Loss of consciousness with collapse
17	75	Female	Lives alone	Yes -metastatic endometrial cancer	Complications related to cancer treatment
18	93	Male	Lives alone, with care visits from son	No	Coryzal illness
19	87	Female	Unclear if lives alone. Daughter involved in care	No	Anti-coagulation complications -known cardiac disease Deterioration in mobility
20	87	Female	Lives alone	No	Likely complications from pneumonia

### Analysis

Five themes are discussed here relating to older hospitalized participants’ views of ACP: (1) Bespoke planning to holistically support a sense of self, (2) ACP as a socio-cultural phenomenon advocating for older people’s rights, (3) The role of personal relationships, (4) Navigating unfamiliar territory and (5) Harnessing resources. The coding hierarchy outlining themes and subthemes is accompanied in the supplementary material. Findings generated from the same interview series regarding the spectrum of ACP is reported in depth elsewhere [3].

#### Theme one: Bespoke planning to holistically support a sense of self

ACP was described as a process that should be bespoke to provide holistic care for older persons in later life. Participants believed that for ACP to fully support an older adult’s identity, discussions and plans should be tailored to each unique person and their circumstances:

*“… judge it according to the person that you’re dealing with” (Participant 2*)

Participants also discussed the need for plans to be sufficiently flexible and dynamic to allow individuals to change decisions at pace if necessary:

*“I think you’ve got to consider updating because things are moving so fast now*, *aren’t they*? *Because what happened the day before yesterday is inclined to be a bit old”*. *(Participant 1*)

Motivations to start ACP were shaped by personal life stories. Participants recounted how experiences of caring for a loved one during serious illness influenced their views. One participant described how experience of end-of-life discussions with her critically ill husband prepared her own planning:

*“…when my husband had stomach cancer…the head of Intensive Care had these discussions with him*, *and I felt quite happy to do that… and I think that’s why I made that with my son because that would be very difficult for him to agree to end of life*…*” (Participants 6*)

For some, a lack of knowledge about loved ones’ plans highlighted the personally sensitive nature of care planning, need for privacy and self-determination. One participant recognised autonomy within ACP as her own husband was initially secretive about his “do not resuscitate” order:

*“My husband had that*, *do not resuscitate…he died of cancer…and I didn’t even know he’d done that ‘til the doctor had mentioned it*, *“Did you tell your wife*? *…he said*, *“Why*? *That’s my business…That’s what I want…and that was the end of it…” (Participant 13*)

Whilst many participants appreciated the role of ACP for themselves, a few had limited or no experience of ACP. Some believed that ACP was irrelevant or unnecessary for their own life. The dismissive attitude towards ACP resulted in a lack of formal planning for their health and wellbeing:

*“Well I haven’t got any experience myself…it’s been very interesting*… *but more on other people*… *I’m fortunate*, *I’m lucky*, *I’m not in that sort of group*.*” (Participant 20*)

Those who were keen to engage with ACP expressed a preference for it to support the natural rhythms of life. Many spoke about the need to maintain a sense of normality and focus plans on the individual’s personal preferences around health and social care. Medicalising ACP by involving doctors was deemed unnecessary by some participants:

*“Well*, *I don’t see why the doctors should be involved” (Participant 18*)

Participants hoped that ACP could prevent institutionalisation through appropriate home support:

*“Well*, *my personal care plan would be if I can’t help myself*, *I wouldn’t want to go into a home…I’d rather go home to me (sic) own family…” (Participant 9*)

The timing of discussions around ACP was an important factor in its uptake. Many expressed the importance of the right place, person and pace in successful discussions:

*“…don’t be too precipitous*. *Let things take their own course and go at their own speed…” (Participant 16*).

In summary, participants expressed a need for ACP to holistically support their sense of self by respecting their individuality, and honouring their life narrative, personal wishes, and preferred way-of-life.

#### Theme two: ACP as a socio-cultural phenomenon advocating for older persons rights

Participants perceived ACP to be a socio-cultural phenomenon, with wider socio-cultural factors influencing ACP, and reciprocally, ACP having an impact on society. Participants believed that ACP helped to advocate for the social rights of older person, helping to create a better future. For example, one participant believed that that planning future care would improve care within society as a whole:

*“Well*, *future care planning is if you’ve got to have a better future*, *care planning more than what we have today…to give us the best we can get*…*” (Participant 19*)

Participants thought including advice on financial benefits within ACP might help older persons receive “whatever help they can get” (participant 20).

Some participants believed that ACP discussions are universally relevant for later life. For example, one participant expressed that everyone should be given the option of discussing their cardio-pulmonary resuscitation status:

*“I think people should always be given the opportunity to discuss it*, *but some people again probably would not want to do it…and that should be accepted*.*” (Participant 17*)

ACP was described as transcending generations, communities, and organisations, influenced by law and the government. This was particularly important for isolated, incapacitated individuals:

*“…if they can’t do for themselves*, *they cannot do nothing (sic)…who will care for you*, *if he doesn’t have any family or he doesn’t have any friends*, *it’s left to the Government*, *the GP*, *all them people (sic) and the nurses and to care for you*.*” (Participant 19*)

Culturally, some individuals were aware of spiritual and religious influences on ACP. Others were influenced by the media; one participant reflected on advance decisions to refuse treatment after hearing news coverage on motor neurone disease:

*“…that case recently …that man with motor neurone disease… it’s a horrible thing and I’m not sure that I wouldn’t want to be able to say that I’ve had enough*…*I can’t do this…” (Participant 8*)

Participants felt that it was important for ACP to protect and uphold the rights of older adults from abuse and discrimination, particularly safeguarding vulnerable or isolated individuals. Concerns were raised about the need to protect individuals’ privacy and prevent fraud in online ACP. Participants strongly advocated for control, transparency, and safeguards over the use of personal data within EPaCCs. Consent and restricting access to highly sensitive information regarding personal health and circumstances was important:

*“I’d be very cautious about that…general accessibility without control really” (Participant 7*).

Some participants believed that ACP in hospitals might improve safety and reduce neglect of older persons during transitions of care:

*“They should look into people’s circumstances when they discharge them late at night… there’s a lot of people that are on their own*. *They’re more vulnerable*, *aren’t they*?*” (Participant 20*)

Older adults believed that an inclusive approach should be taken to protect social rights, particularly advocating for those with disabilities. One participant was unable to read the Coordinate My Care leaflet due to being visually impaired and he stressed the importance of accessibility in ACP:

*“Writing that can’t be read by half of the population–those are the people who are supposed to be reading this stuff”*. *(Participant 7*)

Others highlighted the need to avoid ageism. One participant struggled with condescending stereotypes of what older people’s needs are within ACP:

*“… accept patients as they are*, *I find some people…do talk down to the patient particularly when you’re older and I find that very difficult and actually very offensive sometimes”*. *(Participant 17*)

Altogether, participants described ACP as being shaped by societal and cultural influences whilst impacting on society itself, with the positive effect of upholding older citizen’s needs and rights.

#### Theme three: The role of personal relationships

Participants described the importance of building up a supportive social and healthcare network for both medical care and social functioning. Social isolation was described as a challenge to good health and social care in later life. Several participants felt that ACP was particularly relevant to support isolated individuals, with older individuals who live on their own requiring ACP, rather than those who have supportive social networks:

*“Well*, *I just think advanced care plan*, *I would think that is [for] somebody who lives on their own…” (Participant 13*)

Participants suggested that there was a symbiotic relationship between ACP and supportive networks. Networks were critical for the success of ACP, and foresight with ACP was needed to establish supportive networks to benefit older persons. One participant described that lack of supportive networks was problematic:

*“…people that just cannot cope anymore; their mobility’s gone*, *or they might have a slight mental problem or whatever or they’ve got no family to pop in and out and look after them*.*”(Participant 14*)

Although some discussed the impact of isolation, others discussed the role of friends, family, and trusted contacts within ACP. First degree relatives or partners, particularly spouses, were often listed as power of attorney. Care plans were often viewed as tools to help loved ones make decisions about care, rather than clinicians:

*“I don’t think those people come into it*. *It’s the nearest family to you I think*.”

As care plans were to be enacted by participants’ loved ones, several believed that the contents of a care plan should be disclosed to those closest to them:

*“[Do not attempt resuscitation (DNAR) orders] should be discussed with all the family…who come in contact with them…"(Participant 9*)

Others acknowledged that older persons might wish to shield their family from information maintaining strict confidentiality:

*“Oh please*, *don’t tell my children what’s wrong with me*. *I know what’s wrong with me*. *I’ve got cancer and I don’t want them to know*. *I don’t want them to get upset*.*” (Participant 2*)

Many participants were aware of how complex family dynamics might impact on ACP and the discussions around it. One participant stressed the need for the GP to explore family dynamics during ACP with older participants:

*“… the GP … should*, *in a very informal way*, *find out as much as possible about the patient and his or her family because some families are very free and easy with each other*, *and some have hidden dramas in the background which can alter the situation quite dramatically*.*” (Participant 16*)

In some cases, complex family relationships resulted in a greater need to engage with ACP to avoid conflict. For example, one participant stated that nominating a power of attorney had been driven by wishing to avoid family rifts:

*“I’ve got two sons and they don’t get on very well because they’ve got different religious reasons… I didn’t want there to be any argument…so I’ve made you know power of attorney to different people”*. *(Participant 6*)

In addition to the role of ACP in communicating preferences to loved ones, communication between participants and their health and social care team was a central function of ACP. One participant valued online ACP portals for older adults, enabling a shared, transparent communication with care teams:

*“…I think that’s probably quite important …otherwise there might be currents going that they would think inadvisable…it’s better if everybody is singing from the same hymn sheet…” (Participant 10*)

In summary, participants viewed ACP as being influenced by connections with others at multiple levels, including personal relationships or lack of them, together with health and social care networks. In most cases, engaging with ACP had a protective role for established relationships.

#### Theme four: Navigating unfamiliar territory

Participants believed that ACP in later life involved contemplating the unknown. Due to the potential lack of knowledge around the process of ACP, participants highlighted the need to make ACP accessible, including the language and approach. Although some participants understood the terminology surrounding ACP, others were unfamiliar, feeling confused by jargon:

*“you don’t take it in because you’re well…You don’t take any of this in until you need a doctor or a hospital… I have heard of it*, *but it just goes in and goes out” (Participant 3*)

It was important for doctors to avoid overwhelming patients with excessive questions during ACP:

*“You can keep on asking*, *asking*, *asking questions…but erm there comes a point really when I think you overload people with too much information… so they might eventually all turn their nose up and say*, *“no*, *thank you*!*” (*Participant 10)

Participants felt that the unpredictability of the future was reflected in the uncertainty of ACP. For example, one participant concluded that ACP can only be a basic outline due life’s unpredictability:

*“…I have got a very rough-and-ready embryo care plan already going in that I’ve saved for a rainy day*. *Not gone into any more detail than that*. *Not knowing what kind of rain it’s going to be*, *which direction it’s going to come from*.*” (Participant 16*)

One participant pointed out that serious illnesses such as cancer cannot be predicted, making planning impossible:

*“it’s reactive fundamentally if something goes wrong with your health then you take action…I can’t plan for the fact that I’m going to get cancer…so there’s no advance planning… you don’t know what the future is”*. *(Participant 4*)

Facilitators to navigating ACP included clinicians receiving training, familiarising themselves with patients’ needs and readiness to discussions. One participant believed that checking individual’s preparedness to discuss the natural history of chronic illnesses was essential when initiating ACP:

*“The opportunity should be offered to say…this particular illness does change over the period of time and different things could happen…would you like more information…sort of given the option …if you don’t want to talk now it’s fine…if you feel you do in a couple of weeks’ time*, *we’ll go for it*.*”(Participant 1*7)

After reflecting on Coordinate My Care, one participant valued the sense of preparation that ACP brings into people’s lives and the importance of preparing for the discussion itself:

*“It’s being prepared really…like Boy Scouts*. *You’ve got to start somewhere*, *but there’s no good jumping into the middle*…*They haven’t spoken to you about it and they meet you one day and they say*, *“what do you think” and they go Helter Skelter*, *into all these things and you think to yourself “what on earth are they talking about”*. *(Participant 1*)

In summary, older participants believed that ACP involved navigating unfamiliar territory. Where possible, participants believed it was important to attempt to prepare for discussions, using easy-to-understand language, assessing an individual’s readiness to plan future care, and acknowledging the inherent unpredictability of life, particularly when facing serious illness.

#### Theme five: Harnessing resources

Appropriately harnessing resources was essential to engage with and ultimately, enact ACP. Resources included patient information, time, health and social care staffing and financial investment in health and social care. Participants recognised information within ACP as a precious resource to be harnessed appropriately. Although some individuals wished to limit self-knowledge regarding their medical condition, others viewed knowledge of their illness as crucial in making treatment decisions:

*“I think it’s useful to know the prognosis if there are any you know issues riding round it… you’re going to die in 6 months but if we operate you might get another month or something…” (Participant 6*)

There was an expectation to appropriately document and communicate health records, sharing appropriate information to manage physical health:

*“And if you have someone keeping an eye on you*, *information about your medical condition is probably good for them to know about as well” (Participant 12*)

Concerns over data security were a barrier to EPaCCS. However, provided that data was restricted to appropriate contacts, many participants showed a positive attitude to Coordinate My Care, believing it increased interoperability between different care settings, reduced bureaucracy and provided clinicians with key information during emergencies:

*“…everybody’s in the loop*, *everybody; the ambulance people*, *the doctors*, *the social workers…so everybody knows what’s going on…so she can’t escape and being left unattended…” (Participant 20*)

Participants described links between ACP, financial and human resources. Some believed that ACP meant personal financial planning and making a will. Participant 11 believed that “*handling matters financial*, *administrative and so on…”* was a benefit of ACP. Others believed that money was an enabler to care, and poverty was a barrier to care in ACP:

*“… money can’t buy everything but having money available means that I can get care from a variety of sources*, *which I couldn’t get if I didn’t have any*.*” (Participant 16*)

Participants recognised the limits of a stretched healthcare system as relevant to ACP. There was a need for pragmatism in realising what could be afforded, focusing on those most in need:

*“I guess money is a big one and recognising what is realistic in terms of care planning…I think it is just being realistic … trying to catch people in the net who really do need to be caught in the net*.*” (Participant 17*)

Some participants doubted whether healthcare professionals had the time for ACP, particularly due to staff shortages. For example, one participant doubted whether Coordinate My Care would work because GPs are stretched for time:

*“I can’t see the GPs the doctors are going to be happy with this when they’re pressed for time…” (Participant 6*)

In summary, older participants believed that ACP involved harnessing resources effectively. This included handling patient-sensitive data responsibly and having an awareness of financial and staffing issues in a healthcare system the participants believed was frequently under pressure.

## Discussion

Participants in this study had lived experience of being acutely hospitalized in later life. This analysis suggests that maintaining and supporting their identity during ACP, including respect for their personal life, lifestyle, and rights as individuals was highly valued. To engage with ACP, participants highlighted the importance of a supportive system of relationships within wider society, influenced by relationship dynamics, communication styles and societal frameworks. Furthermore, older persons considered the challenges of navigating the uncertainties of planning for the future, harnessing information with potentially strained resources. Altogether, these themes demonstrate that maintaining a sense of self during the process of ACP in later life is dependent on relationships within wider society and resourcefully navigating an unpredictable future.

Our previous paper explained how an emergency hospitalisation in later life can act as a catalyst for ACP [3]. As the quantitative element of the mixed methods paper had revealed that one in five patients aged 70 and over were likely to be in the last year of life, the qualitative element explored how an emergency hospitalisation in later life can be used as an opportunity to initiate advance care planning [3]. The novel results presented in the current paper have a different focus and explore how ACP impacts the lives of older persons, including consideration for how ACP can be enacted for older persons within society, including from a health systems perspective. To our knowledge, it is the first qualitative study to report in-depth views of older patients who have been acutely hospitalised towards the use of Electronic Palliative Care Coordination Systems (EPaCCS). When combined, these two papers give detailed insights into when ACP is likely to be needed, how it can be initiated and the impact it may have over time.

Older hospitalized patients expressed a need to holistically support a sense of self when planning for future care. Participants wished to maintain independence and control over the content of ACP, for decisions to acknowledge their personal situation, life story and lifestyle. Participants believed ACP needed to be flexible, bespoke to the person and situation, as shown in other studies where the iterative nature of ACP is important [12, 30]. To date, the emphasis on preservation of identity in ACP is particularly well documented in dementia care [12, 31] and end-of-life care literature, placing emphasis on autonomy and dignity [32]. This analysis provides evidence that preserving the “self” is also relevant to a general older inpatient population after an acute illness.

Planning future care was viewed not only as a medical issue but as a social phenomenon by participants, influenced by relationships and society. Family support and personal relationships were key. This complements research in frail older adults experiencing acute illness, where future care preferences, such as place of care, were shaped by availability of supportive contacts [33]. Research elsewhere shows ACP amongst older seriously ill inpatients can involve familial substitute decision makers [14]. This study supports ACP clinical recommendations which advise considering social circumstances, carers and family [34], including discussing goals of care in family meetings [35]. It backs evidence demonstrating that socio-cultural [36] and religious influences [37] can affect discussions. Prioritising communication between patients, families and clinicians during ACP is key [38]. However, the epidemic of loneliness in the ageing population [39] poses a challenge to ACP. Social isolation has been associated with reduced engagement in ACP and might require targeted efforts to boost uptake [40].

This analysis outlines ACP as a ‘journey’ traversing uncertainty and unfamiliarity, with a need to attempt to prepare for future care by harnessing information and accessing resources. Findings elsewhere suggest that uncertainty about future care may variably persuade patients to discuss ACP after hospital discharge [27] and that uncertainty shapes patient’s experiences and future care priorities in advanced disease [41]. Interviews in end-of-life care also show that “preparedness” is valued, including information for decision-making [32]. Information is also crucial in the emergency setting for patients with palliative care needs and their clinicians [42]. Our participants believed that information within ACP is a key resource to safeguard, potentially being sensitised to concerns about data security in the wake of previous cyberattacks [43].

In preparation for ACP, participants perceived a tension between the idealism of ACP strategies for older adults, with the realities of care provision for vulnerable or isolated persons, within a financially stretched and understaffed system. Similar concerns are voiced by carers of older hospitalized adults [2]. As such, participants were cognisant of the challenges of health inequalities [44] and the cost of health and social care in an ageing population [45]. Research investigating the economic implications of ACP supports these concerns [46–49]. However, there is increasing attention regarding potential financial savings from ACP through optimising healthcare resources use [46, 47]. Overall, participants welcomed practical support and advice on accessing resources when planning future care in later life.

### Clinical applications

This study gives rise to recommendations from older patients to clinicians and commissioners on ACP following an emergency hospitalization and more widely. Whilst patients expressed positive attitudes to ACP, the process should respect their identity and social circumstances. The importance of preserving self-identity gives credence to healthcare professionals’ warnings against a “tick-box approach” [5]. The findings support shared decision-making and person-centred care during ACP [8, 50, 51]. Uncertainty and frustration with ACP jargon by participants sends a powerful message to clinicians to use clear, accessible language and check understanding during discussions. Co-designing approaches to facilitate ACP discussions with older adults may prove beneficial in this regard.

The ability to share up-to-date information between care settings following an emergency using EPaCCS was highly valued by patients. The findings reinforce the importance of EPaCCS such as Coordinate My Care, particularly in flexibly updating information and improving concordance in ACP decisions, including preferred place of care and death [7]. Maintaining control and transparency in how personal healthcare data is used within EPaCCS was key to participants in this study. Therefore, ACP must have robust data protection measures in place [52] -older patients may require reassurances regarding the cybersecurity and confidentiality of EPaCCS to improve its uptake.

### Strengths and limitations of the study

This study provides previously overlooked views of ACP from older patients experiencing an emergency hospitalization, which may be considered a “difficult to reach population” [16, 33]. A wide range of views were gathered, since patients were not excluded based on any diagnosis and were admitted to both medicine and surgery. Acutely hospitalized older adults may be a cohort to particularly benefit from improvements in ACP. Collaborating with a panel of patients and carers with an interest in ACP in the co-design of topic guide strengthened the study [23].

The time-pressured acute hospital environment potentially limited the depth of interviews. The opinions of patients affected by dementia are underrepresented, since appropriate mental capacity was a pre-requisite for interview. The study also lacks insights from patients treated in critical care, as they were excluded for being medically unstable to interview. It has been previously reported that clinicians may be paternalistic and protective during the recruitment phase of research [53]. Whilst the use of the randomised patient list helped to systematically identify which patients to approach for participation, relying on the inpatient clinical team to assess whether patients were stable for interview may have limited the study through introducing bias via a possible gate-keeping effect. The interviewer’s medical and research background may have potentially influenced interpretation of the findings towards a more “medicalised” paradigm, although this was discussed in an interdisciplinary context. Further work should incorporate the study findings to co-design an approach to facilitate ACP in later life, alongside older persons and carers.

## Conclusions

Older persons who had been hospitalized as an emergency viewed ACP within the context experiences and challenges, both in their personal life and in wider society. Holistically supporting a sense of personal identity is important following acute illness, supported by a network of relationships. Adequate practical preparation for planning future care through harnessing information and resources is required, despite the uncertainties that lie ahead. Consequently, EPaCCS may have a supportive role in this context in sharing up-to-date ACP information during transitions of care following emergency hospitalization.

## Supporting information

S1 File Semi-structured interview schedule. (DOCX)

S2 File Coding hierarchy-themes and subthemes. (DOCX)

S3 File Consolidated criteria for reporting qualitative studies (COREQ): 32-item checklist. (DOCX)
