# Supplementary material for: Views of advance care planning in older hospitalized patients following an emergency admission: A qualitative study
Source: PLoS One. 2022 Sep 1;17(9):e0273894. doi: 10.1371/journal.pone.0273894 (PMC9436063; doi:10.1371/journal.pone.0273894)
Supplement: S1 File — (DOCX) [file pone.0273894.s001.docx]

**SEMI-STRUCTURED INTERVIEW SCHEDULE**

**VIEWS OF ADVANCE CARE PLANNING IN OLDER HOSPITALIZED PATIENTS FOLLOWING AN EMERGENCY ADMISSION**

Questions in bold, prompts below in bullet point format.

The term “future care planning” was used with participants during the interview.

**PART ONE**

**PATIENT’S BACKGROUND AND IMPLICATIONS OF LANGUAGE TO PATIENT**

**1) Let’s start by talking a bit about your background…**

**Prompts**

- Tell us a bit about why/how you have come into hospital
- How are you feeling?
- Age
- Tell us about your social background: Do you have carers/live alone?

**2) We are here to talk about “future care planning”. You may have not heard of this term before as it might be new to you and there is no “right answer”.**

**What comes to mind when you hear the term “future care planning”?**

**3) You may or may not have heard some of these terms. It may seem like a lot of terms -they might be new to you and there are no “right answers” (show list).**

**What do you think of they might mean? How do you feel about these terms?**

**Advance care planning**

**Care plan**

**Social care plan**

**Medical care plan**

**Urgent care plan**

**Personal care plan**

**PART TWO: PERSONAL SIGNIFICANCE TO PATIENTS –POSITIVES AND NEGATIVES**

**4) Have you any previous experience of care planning/future care planning?**

**Prompts**

- Do you have a future care plan (e.g. Coordinate My Care or a local GP care plan)?
- Do you know anyone with a future care plan?
- Has your doctor or anyone spoken to you before about future care planning?
- Do you have a power of attorney?
- Do you have a living will?

**5) How might future care planning work in someone’s lifestyle? / What might the potential benefits of future care planning be?**

**6) How would you like future care planning discussed with you? How can we make this acceptable to older adults?**

**Prompts**

- Should you start the discussion or should doctors/medical staff start the discussion?
- Would you mind this being raised in hospital or at home? Why?
- How would you like this topic raised in hospital?

**7) Do you think there might be possible problems with future care planning?**

**8) Is there anything that would put you off answering questions about care plans or be off-putting about the process?**

**PART THREE: CONTENT OF FUTURE CARE PLANS**

**We would like to discuss your ideas about care planning**

**9) What do you think should be included in a future care plan?**

NB Suggestions may be spontaneously offered from the patient, so allow for response to this open question, then go through some specific ideas in the next question.

**10) We would like to show you an example of things that might be included in a future care plan^[[1]](#footnote-1)^. Would you expect these in a future care plan?**

Things that are important for you to carry on doing

Values important for you for treatment and care

Things that you do not want in your treatment or care

Concerns for the future

Benefits advice

Information about your medical condition

How your illness might affect you

Prognosis

Key contacts: medical, carers, social support, next of kin (family/friends)

Asking about a person you can trust to speak for you and help make decisions about your health if you cannot do so yourself in the future (a Welfare Attorney)

A living will or advance decision to refuse a specific treatment

Cardiopulmonary resuscitation decisions (DNA CPR); attempting to restart a person’s heart

**Prompts**

- **Should a care plan involve a discussion on these?**
- **Is there anything that should be avoided?**

**12) Do you think it would be good to offer patients a care plan specific to their current illness made with their doctor? This may involve discussing prognosis and what to do in case of future change in health.**

**PART FOUR: SHARING FUTURE CARE PLANS**

**13) Would you discuss a future care plan with a loved one?**

**Prompts**

- Why would you/would you not discuss this with someone?

**14) Who should be able to read your future care plan?**

**Prompts**

- Next of kin
- Carer
- Ambulance crew
- GPs
- Urgent care centres
- Hospital doctors and nurses
- Social services

**15) Would you be happy for a care plan to be on the web on a secure link accessed by password/electronic version?**

**16) This is a patient leaflet for Coordinate My Care which does future care plans in the NHS. What do you think?**

**Prompts**

- Is there anything you like about this?
- Is there anything you don’t like about this?

1. List of potential topics within a future care plan adapted from Boyd K et al. Advance care planning for cancer patients in primary care: a feasibility study. Br J Gen Pract 2010 449-458 [↑](#footnote-ref-1)
