# Supplementary material for: Views of advance care planning in older hospitalized patients following an emergency admission: A qualitative study
Source: PLoS One. 2022 Sep 1;17(9):e0273894. doi: 10.1371/journal.pone.0273894 (PMC9436063; doi:10.1371/journal.pone.0273894)
Supplement: S2 File — (DOCX) [file pone.0273894.s002.docx]

**Coding hierarchy -themes and subthemes**

| Theme | Subtheme |
| --- | --- |
| 1. Bespoke planning to holistically support a sense of self | 1. ACP bespoke to the person and situation 2. ACP shaped by memories of a personal life story 3. Maintaining the rhythms of life |
| 1. ACP as a socio-cultural phenomenon advocating for older people’s rights | 1. ACP as a socio-cultural phenomenon 2. Safeguarding the rights of older adults |
| 1. The role of personal relationships | 1. Connectedness 2. Familial roles and relationships 3. Continuums of communication |
| 1. Navigating unfamiliar territory | 1. Crossing unfamiliar paths 2. Making the best of it -being prepared for ACP |
| 1. Harnessing resources | 1. Harnessing information as a previous resource for ACP 2. Navigating finances and scarcity of resources |
