# Supplementary material for: Views of advance care planning in older hospitalized patients following an emergency admission: A qualitative study
Source: PLoS One. 2022 Sep 1;17(9):e0273894. doi: 10.1371/journal.pone.0273894 (PMC9436063; doi:10.1371/journal.pone.0273894)
Supplement: S3 File — (DOCX) [file pone.0273894.s003.docx]

**Consolidated criteria for reporting qualitative studies (COREQ): 32-item checklist**

Developed from:

Tong A, Sainsbury P, Craig J. Consolidated criteria for reporting qualitative research (COREQ): a 32-item checklist for interviews and focus groups. *International Journal for Quality in Health Care*. 2007. Volume 19, Number 6: pp. 349 – 357

**YOU MUST PROVIDE A RESPONSE FOR ALL ITEMS. ENTER N/A IF NOT APPLICABLE**

| **No. Item** | **Guide questions/description** | **Reported on Page #** |
| --- | --- | --- |
| **Domain 1: Research team and reﬂexivity** |  |  |
| *Personal Characteristics* |  |  |
| 1. Inter viewer/facilitator | Which author/s conducted the interview or focus group? | Materials and procedure (pp 6 of manuscript  -AMB (first author) |
| 2. Credentials | What were the researcher’s credentials? E.g. PhD, MD | MSc (first author) co-working with academics with PhD/MD  Listed on original title page (submitted 5.11.21) |
| 3. Occupation | What was their occupation at the time of the study? | Materials and procedure (pp 7 of manuscript  -Medical doctor with a research interest in advance care planning |
| 4. Gender | Was the researcher male or female? | Materials and procedure (pp. 7 of manuscript)  -interviewer was female |
| 5. Experience and training | What experience or training did the researcher have? | Materials and procedure (pp. 7 of manuscript)  Data analysis (pp. 8 of manuscript)  Interviewer is a trained medical doctor. Research team includes CEU (Professor and Consultant in Palliative Medicine) and SA (Health Psychologist with expertise in qualitative methodology). |
| *Relationship with participants* |  |  |
| 6. Relationship established | Was a relationship established prior to study commencement? | Materials and procedure (pp. 7 of manuscript)  No -the researchers were not part of the usual clinical care team |
| 7. Participant knowledge of the interviewer | What did the participants know about the researcher? e.g. personal goals, reasons for doing the research | Materials and procedure (pp. 7 of manuscript)  Participants aware that researcher (AMB) was clinically trained doctor and academic interest in advance care planning |
| 8. Interviewer characteristics | What characteristics were reported about the inter viewer/facilitator? e.g. Bias, assumptions, reasons and interests in the research topic | Strengths and limitations of the study (pp. 25 of manuscript)  The interviewer’s medical and research background may have influenced interpretation of the findings towards a more “medicalised” paradigm, although this was discussed a team to provide an interdisciplinary context. |
| **Domain 2: study design** |  |  |
| *Theoretical framework* |  |  |
| 9. Methodological orientation and Theory | What methodological orientation was stated to underpin the study? e.g. grounded theory, discourse analysis, ethnography, phenomenology, content analysis | Data analysis (pp. 8 of manuscript)  -Thematic analysis |
| *Participant selection* |  |  |
| 10. Sampling | How were participants selected? e.g. purposive, convenience, consecutive, snowball | Participants and recruitment (pp. 5 of manuscript)  Recruited consecutively according to a random computer-generated list |
| 11. Method of approach | How were participants approached? e.g. face-to-face, telephone, mail, email | Materials and procedure (pp 6 of manuscript)  Participants approached in person by the researcher, following initial check from a member of the clinical team |
| 12. Sample size | How many participants were in the study? | Results  (pp. 8 of manuscript)  -20 participants |
| 13. Non-participation | How many people refused to participate or dropped out? Reasons? | Results  (pp. 8 of manuscript)  -No dropouts following consent. |
| *Setting* |  |  |
| 14. Setting of data collection | Where was the data collected? e.g. home, clinic, workplace | Materials and procedure (pp 7 of manuscript)  -Inpatient hospital setting |
| 15. Presence of non-participants | Was anyone else present besides the participants and researchers? | Materials and procedure (pp 7 of manuscript)  A functioning inpatient hospital ward setting was used for interviews. |
| 16. Description of sample | What are the important characteristics of the sample? e.g. demographic data, date | Participants and recruitment  (pp. 5 & 6 of manuscript)  Demographic data included -older persons aged 70+ with appropriate mental capacity and currently experiencing an emergency hospitalization |
| *Data collection* |  |  |
| 17. Interview guide | Were questions, prompts, guides provided by the authors? Was it pilot tested? | Materials and procedure (pp 7 of manuscript)  Questions were provided by a semi-structured interview guide pilot tested by the research team and a group of patients and carers with an interest in urgent care planning |
| 18. Repeat interviews | Were repeat inter views carried out? If yes, how many? | Materials and procedure (pp 7 of manuscript)  Each of the 20 participants was only interviewed once, with an average length of 34 minutes. |
| 19. Audio/visual recording | Did the research use audio or visual recording to collect the data? | Materials and procedure (pp 7 of manuscript)  Digital audio-recording used |
| 20. Field notes | Were ﬁeld notes made during and/or after the inter view or focus group? | Materials and procedure (pp 7 of manuscript)  Field notes were made by the interviewer. |
| 21. Duration | What was the duration of the inter views or focus group? | Materials and procedure (pp 7 of manuscript)  The average length of the interview was 34 minutes. |
| 22. Data saturation | Was data saturation discussed? | Materials and procedure (pp. 7 of manuscript)  Data collection continued until thematic saturation was achieved. |
| 23. Transcripts returned | Were transcripts returned to participants for comment and/or correction? | N/A |
| **Domain 3: analysis and ﬁndings** |  |  |
| *Data analysis* |  |  |
| 24. Number of data coders | How many data coders coded the data? | Data analysis (pp. 8 of manuscript)  All transcripts coded by AMB. 10% of transcripts were dual coded (GS and AMB). Subthemes and themes discussed with 2 additional researchers (CEU and SA) |
| 25. Description of the coding tree | Did authors provide a description of the coding tree? | Refer to coding hierarchy (themes and sub-themes) in supplementary material S2 |
| 26. Derivation of themes | Were themes identiﬁed in advance or derived from the data? | Data analysis (pp. 8 of manuscript)  Themes were derived from data. |
| 27. Software | What software, if applicable, was used to manage the data? | Data analysis (pp. 8 of manuscript)  Data were manually coded (using Microsoft Word), specialist software not applicable for this study. |
| 28. Participant checking | Did participants provide feedback on the ﬁndings? | N/A |
| *Reporting* |  |  |
| 29. Quotations presented | Were participant quotations presented to illustrate the themes/ﬁndings? Was each quotation identiﬁed? e.g. participant number | Included in the analysis section of results throughout.  Yes -quotations presented and identified according to participant number. |
| 30. Data and ﬁndings consistent | Was there consistency between the data presented and the ﬁndings? | Included in analysis and discussion sections.  Data was consistent with findings -discussed |
| 31. Clarity of major themes | Were major themes clearly presented in the ﬁndings? | Included in the analysis section of results throughout with summary on pp. 10 of manuscript  Yes, major themes outlined, described, and discussed. |
| 32. Clarity of minor themes | Is there a description of diverse cases or discussion of minor themes? | Included in the analysis section of results throughout.  Subthemes and diverse cases discussed in report |

**Once you have completed this checklist, please save a copy and upload it as part of your submission. When requested to do so as part of the upload process, please select the file type: *Checklist*. You will NOT be able to proceed with submission unless the checklist has been uploaded. Please DO NOT** **include this checklist as part of the main manuscript document. It must be uploaded as a separate file.**
